# Diversity in the pathway from medical student to specialist in the Netherlands: a retrospective cohort study

**DOI:** 10.1016/j.lanepe.2023.100749

**Published:** 2023-10-12

**Authors:** Lianne Mulder, Anouk Wouters, Eddymurphy U. Akwiwu, Andries S. Koster, Jan Hindrik Ravesloot, Saskia M. Peerdeman, Mahdi Salih, Gerda Croiset, Rashmi A. Kusurkar

**Affiliations:** a Amsterdam UMC Location Vrije Universiteit Amsterdam, Research in Education, De Boelelaan 1118, Amsterdam, the Netherlands; b LEARN! Research Institute for Learning and Education, Faculty of Psychology and Education, VU University Amsterdam, the Netherlands; c Amsterdam UMC, Vrije Universiteit Amsterdam, Epidemiology and Data Science, Amsterdam Public Health, De Boelelaan 1117, Amsterdam, the Netherlands; d Department of Pharmaceutical Sciences, Utrecht University, David de Wied Building, Universiteitsweg 99, Utrecht, the Netherlands; e Amsterdam UMC Location University of Amsterdam, Faculty of Medicine, Department of Medical Biology, Meibergdreef 9, Amsterdam, the Netherlands; f Amsterdam UMC Location University of Amsterdam, Department Neurosurgery, Meibergdreef 9, Amsterdam, the Netherlands; g Amsterdam UMC Location University of Amsterdam, Faculty of Medicine, Teaching and Learning Centre, Meibergdreef 9, Amsterdam, the Netherlands; h Amsterdam Public Health, Quality of Care, Amsterdam, the Netherlands; i Erasmus MC, Division of Nephrology and Transplantation, Department of Internal Medicine, Dr. Molewaterplein 40, Rotterdam, The Netherlands; j University Medical Center Groningen, Wenckebach Institute for Education and Training, Hanzeplein 1, Groningen, the Netherlands

**Keywords:** Student diversity, Physician diversity, Specialist diversity, Inequality of opportunity, Medical workforce, Cohort study

## Abstract

**Background:**

Medical specialist workforces are not representative of the society they serve, partially due to loss of diversity in the path from student to specialist. We investigated which demographic characteristics of bachelor students of medicine (BSM) are associated with becoming a physician and (particular type of) medical specialist; and whether this suggests ‘cloning’ (reproduction of sameness) of the existing workforce.

**Methods:**

We used a retrospective cohort design, based on Statistics Netherlands data of all first-year BSM in 2002–2004 in The Netherlands (N = 4503). We used logistic regression to analyze the impact of sex, migration background, urbanity of residence, parental income and assets categories, and having healthcare professional parents, on being registered as physician or medical specialist in 2021. We compared our results to the national pool of physicians (N = 76,845) and medical specialists (N = 49,956) to identify cloning patterns based on Essed’s cultural cloning theory.

**Findings:**

Female students had higher odds of becoming a physician (OR 1.87 [1.53–2.28], p < 0.001). Physicians with a migration background other than Turkish, Moroccan, Surinamese, Dutch Caribbean or Indonesian (TMSDI) had lower odds of becoming a specialist (OR 0.55 [0.43–0.71], p < 0.001). This was not significant for TMSDI physicians (OR 0.74 [0.54–1.03], p = 0.073). We found a cloning pattern with regard to sex and migration background. Nationwide, physicians with a Turkish or Moroccan migration background, and female physicians with other migration backgrounds, are least likely to be a medical specialist.

**Interpretation:**

In light of equity in healthcare systems, we recommend that every recruitment body increases the representativeness of their particular specialist workforce.

**Funding:**

ODISSEI.

**Research in context**

**Evidence before this study**

Before this study, the male-female distribution of the physician and specialist workforce in The Netherlands (including per specialty) was known to be unequal. However, there was a gap in the knowledge on the diversity of the workforce with regard to other background characteristics. We searched PubMed for studies evaluating the diversity of specialists in The Netherlands. The keywords (“diverse” [All Fields] OR “diversely” [All Fields] OR “diversities” [All Fields] OR “diversity” [All Fields]) AND (“specialist s” [All Fields] OR “specialistic” [All Fields] OR “specialization” [MeSH Terms] OR “specialization” [All Fields] OR “specialist” [All Fields] OR “specialists” [All Fields]) AND (“netherlands” [MeSH Terms] OR “netherlands” [All Fields] OR “netherland” [All Fields]) yielded 274 results. We did not limit the search with regard to start/end dates, language, or other factors. 2 papers were eligible for screening. One study focused on the experiences and motivation of ethnic minority students in medical education. The other focused on performance appraisal of cultural minority physicians. Both studies refer to a Dutch-language document in which it is estimated that 2–4 percent of hospital specialists have a migration background. The document is unclear about how this estimate was arrived at. The present study leveraged the national non-public healthcare professional register and Statistics Netherlands Microdata to study the diversity of physicians and specialists with regard to sex, migration background, urbanity of residence during high school, parental income/assets categories, and having healthcare professional parents.

**Added value of this study**

Our findings show the loss of diversity in the path from medical student to specialist with regard to sex, migration background, urbanity of residence during high school, parental income/assets categories, and having healthcare professional parents. The study is based on a retrospective cohort design, and on a comparison with the entire national healthcare professional register of The Netherlands. Our findings add value to the existing evidence by indicating which clusters of specialties lack representativeness, and on which dimensions of diversity this is the case. Next to that, this study adds value on a theoretical level: we used quantitative data to investigate the qualitative theory of cultural cloning in medicine by Philomena Essed.

**Implications of all the available evidence**

There are several implications for practice, policy and future research. In light of equity in healthcare systems, it is necessary to increase the representativeness of the specialist workforce. This can be done, for example, through inclusive recruitment and hiring procedures for residency training which do not reproduce a pattern of cultural cloning of the existing specialist workforce. In this process, awareness of cultural cloning—and how to avoid it—is essential. Additionally, implementation of a national registration system for applications and selection outcomes for residency training would enable investigation of potential self-selection and bias.

## Introduction

A representative healthcare workforce is crucial for providing the best possible equitable care to all patients.^1^ The Lancet Global Health Commission on High Quality Health Systems noted that health systems should improve equity, as one of the dimensions of quality of care.^2^ However, the current medical specialist workforce does not mirror the diversity of its patient population.^3^, ^4^, ^5^ A consistently observed pattern is the overrepresentation of those from the highest socio-economic status (SES) strata, and the underrepresentation of medical specialists of color.^3^^,^^6^, ^7^, ^8^, ^9^ The gradual loss of diversity in the path from medical student to specialist is referred to as the ‘leaky pipeline’.^3^ It harms the quality of health professions education^6^^,^^10^ and the provision of healthcare to underserved populations,^11^ limits health providers’ careers^12^ and reproduces societal inequities.^13^ However, there is a gap in the literature on how multiple demographic characteristics combined may influence the odds of becoming a medical specialist. Therefore, this study uses a retrospective cohort design to provide an intersectional picture of the leaky pipeline in the context of The Netherlands. By combining a historical dataset of medical students, the complete register of all physicians and specialists in the Netherlands, and a variety of demographic background variables we can map in which steps of the pipeline diversity is lost.

Internationally, within medical schools, significant ethnicity-related differences in clinical skills assessment^14^^,^^15^ and bias on the basis of sex in the evaluation of interns^16^ are known to contribute to the leaky pipeline. Grades may be important in the competition for residency training or a PhD position—which may increase one’s odds of attaining a residency (training) position,^4^ and one’s opportunities in medical career advancement in general.^14^ Similarity bias in hiring decisions for residency training positions is one of the factors coming into play after medical school.^4^^,^^7^^,^^17^^,^^18^ In many contexts, historical and current conditions (e.g., relating to racism, sexism, classism) result in lower levels of socio-cultural and economic capital for underrepresented groups in medicine, including limited access to social networks in medicine, or to knowing the unwritten codes of conduct. This can impact their pathways from medical training to becoming a specialist.^19^

Research has indicated where diversity is lost between childhood and gaining admission to medical school in The Netherlands, resulting in a student population that does not mirror the diversity of their age group nor future patient population.^20^ About 80% of students come from high income backgrounds. Men (29% of students versus 51.2% of age group) and students with (parents with) a Turkish, Moroccan, Surinamese or Dutch Caribbean migration background (<5% of students versus >10% of age group) are underrepresented. An estimated two to four percent of medical specialists in hospitals have a migration background,^21^ compared to 23% of Dutch 16-year-olds and 21% of medical students.^20^ The unequal sex distribution amongst different specialties is widely acknowledged, but socio-economic background or migration background are less thoroughly investigated. This is concerning, since The Netherlands is becoming increasingly diverse with regard to the number and proportion of people who have a migration background. In 2010, 20.3% of inhabitants had a migration background. In 2022, this had increased to 25.2%.^22^ The limited information and estimates available about the diversity in the physician and specialist workforce, suggest that the changes in the society as a whole may not be reflected in the medical workforce. This indicates the need for a nationwide investigation of the leaky pipeline in medicine.

Since there is no national application system for residency training positions, it is unknown whether the (lack of) representativeness of the specialist workforce is due to self-selection in the application process or inequality of opportunity in the selection procedure. With the exception of family medicine, the application procedures for residency training positions are not centralized. Each national specialty’s association and regional residency program group designs their own selection procedures for residency training.^18^ These procedures vary, but traditional job interviews are common, after a preselection based on motivation letters and CVs.^18^ Based on the literature, we hypothesize that *cultural cloning* in the application process and/or selection procedure might be at play. In ‘*Cloning the Physician’*, Philomena Essed^23^ argued how cultural homogeneity is ‘cloned’ in the medical specialist workforce, based on a preference for those who resemble the type of human which already dominates the specialty. The reproduction of sameness is a means of gatekeeping top positions through systemic discrimination against particular groups based on ethnicity, sex, and other factors. The result is homogeneity within the medical workforce, as “a limited number of cultural and physical attributes are selected to serve as primary markers defining who belongs and who does not, who can qualify as a physician and who cannot (really). Thus, otherwise complex human beings are reduced to traits representing the normative image of their profession, thereby suggesting that no other than these normative traits fit best the requirements for the profession” (p. 131). According to Essed’s theory of cultural cloning, there are a number of racially and culturally charged competencies that are considered relevant in the development of medical specialist skills. These include “intelligence, rationality, emotional detachment, high ambition, high competitive drive, and a workaholic mentality prioritizing work above family” (p. 129). The theory states that these competencies are more likely to be ascribed to people who are e.g., white, European, male, and middle class. We used the theory of cultural cloning in the medical profession to inform our analyses of an important part of the leaky pipeline in medicine. We chose it over other possible options, such as the concepts of similarity bias, implicit bias or affinity bias, as these are often regarded as unconscious phenomena residing within the individual. Essed’s theory also highlights the processes which are systemic and institutional that result in a lack of diversity. For example, it states that modes of cultural cloning should be interpreted within the context of a “total framework of historical and societal forces supporting the cultural cloning of normative profiles of physicians and of other top positions for that matter” (p. 129).

Our research questions were: Which demographic background characteristics of first-year bachelor students of medicine are associated with becoming a registered physician? Which demographic background characteristics of physicians are associated with becoming a (particular type of) medical specialist? And do the odds of becoming a (particular type of) medical specialist, based on different demographic background characteristics, suggest a cloning pattern of the existing workforce?

## Methods

### Study design

We conducted a retrospective cohort study using anonymized non-public microdata from Statistics Netherlands with the protocol in the Supplementary Material. We used the SAMPL and STROBE cohort reporting guidelines as statistical and methodological guidelines.

### Study population

#### BSM cohort 2002–2003 & 2003–2004

We created a cohort of all first-year bachelor students of Medicine (BSM) in the academic years 2002–2003 and 2003–2004. These years were chosen based on the realistic number of years it takes to become a medical specialist (six years of medical school, waiting time, work experience, residency training). The shortest residency programs (e.g., family medicine) require three years, whereas the lengthiest (e.g., cardiology, surgery) take six years to complete. Working part-time, doing a PhD and/or taking parental leave, extend this length.

The original Statistics Netherlands dataset containing all student registrations in The Netherlands only specifies the number of years a student is registered at a particular university. It does not indicate whether a student has enrolled in different study programs in different academic years. This means that students who were enrolled in a different program before Medicine at the same university could not be identified in the original dataset. For example: a university student who started in 2001–2002 with the Pharmacy program at University X, but switched to Medicine in 2002–2003, also at University X, cannot be identified as a first-year Medicine student in the original dataset. However, a first-year Pharmacy student at University Y who switched to Medicine at University Z in 2002–2003 or 2003–2004 is included, as they can be identified as a first-year student at University Z. All 4503 students who were traceable in this dataset as first-year Medicine students are included in our cohort. No students were excluded.

The pseudonymised non-public healthcare professional register (‘*BIG register’*) of 2021 was used to determine which BSM had become a registered physician and medical specialist. Physicians are legally required to have a BIG registration in order to practice and begin residency training. In The Netherlands, a physician is anyone who is currently registered as such after having completed medical school. ‘Specialist’ is the term used for physicians who have completed their residency training and are registered within a particular specialty in the national register of healthcare professionals. Other countries may refer to this group as ‘attending physicians’.

#### All physicians & specialists in 2021

Using the same BIG register, we compared our cohort population to the national pool of physicians and medical specialists with an active registration. We identified the three clusters of The Royal Dutch Medical Association’s Medical Specialties Council (Appendix 1). Cluster 1 consists of family, elderly care and intellectual disability medicine. Cluster 2 consists of all hospital specialties. Cluster 3 is comprised of occupational, insurance, and public health medicine.

#### Data of small groups & combination of specialties

Statistics Netherlands prohibits disclosure of group sizes (including within-variable categories) smaller than 10. We therefore replaced frequencies between 0 and 4 by ‘<5’ and frequencies between 5 and 9 by ‘<10’. Their respective regression results were replaced by ‘Hidden’.

### Variables

Variables in the study are described in Table 1. Outcome measures are: did a person become a physician (no/yes); and did the person become a (particular type of) specialist (no/yes).

**Table 1:** Demographic data recorded for each student and their parents.

Variable	Values	Rationale for categorization	Rationale for selecting variable
**Student data**			
Sex	0 = Male 1 = Female^a^		The known male:female ratio of BSM of 3:7^19^
Migration background	Statistics Netherlands categorizes migration background based on someone’s country of birth, and/or that of their parents. If one parent was born in the Netherlands and the other abroad, the country other than the Netherlands is chosen. We recoded all country codes in 5 groups: 0 = Dutch (no migration background), 1 = Turkish/Moroccan 2 = Surinamese/Dutch Caribbean/Indonesian 3 = European (defined as EU/EEA/Switzerland) 4 = Other migration background Due to small sizes of certain groups, we combined groups 1 & 2 (abbreviated as TMSDI); and groups 3 & 4 (called ‘Other’), in a number of regression analyses. The descriptive statistics are, wherever permitted within the regulations around group sizes, given in as much detail as possible to enable better interpretation.	The differences in types of migration history of migrant populations.	The known inequalities which students with a migration background face in e.g., the selection procedure for medical school^19^ and in clinical assessment and grading.^15^ Research shows unequal opportunities in moving up from physician to specialist,^7^ regardless of whether they hold a PhD or not.^4^
Degree of urbanity of postal code, of address at age 16	Based on the average number of addresses per km^2^ of the area: 1. Very strong (2500 or more) 2. Strong (1500–2499); 3. Average (1000–1499); 4. Weak (500–999); 5. Not at all (less than 500)	Categorization by Statistics Netherlands	The known shortage of healthcare providers in rural areas.
**Parental data**			
Income percentile of parent with highest income	Scale of 0–100, categorized for regression analysis: 0 = Percentiles 1–70 1 = Percentiles 71–90 2 = Percentiles 91–100	Best possible balance in each category	The known barriers of low SES in the medical education field, and the disproportionate share of students from high-income families among HPE students.^19^ Income and assets percentiles, rather than their values in euros, were included because percentiles indicate the relative position one occupies compared to the rest of the population.
Assets percentile^b^	Scale of 0–100, categorized for regression analysis: 0 = Percentiles 1–40 1 = Percentiles 41–80 2 = Percentiles 81–100	Best possible balance in each category	
Number of parents who are registered healthcare professionals	0, 1, 2, categorized for regression analysis: 0 = 0 parents 1 = 1 or 2 parents	Best possible balance in each category	The known influence of having a network in the medical field as a facilitator in the route from childhood to practising medicine.^19^^,^^24^

a It is acknowledged that not every individual is ‘male’ or ‘female’, but Statistics Netherlands only has two possible sex categories. This means that e.g., intersex persons either have missing data on their sex registration, or are categorized as male or female.

b The assets percentile concerns e.g., property, shares and savings on the household level. It is often lower than the income percentile due to the influence of (primarily mortgage) debts. When parents live in different households, they may each have a different assets percentile. In that case, we selected the highest percentile.

### Statistical analysis

First, we performed univariable logistic regression analyses on the BSM cohort to examine which of the demographic variables were associated with becoming a physician/medical specialist (regardless of time-to-event period). Statistical level of significance was set at 0.05. We examined data for evidence of multicollinearity amongst the independent variables using both the Pearson correlation coefficients between variables, and the variance inflation factor (VIF) of each variable. Second, we used multivariable logistic regression to build association models, to investigate whether certain groups of individuals had higher odds of becoming a physician or (particular type of) medical specialist than others. Third, we combined the results of the multivariable logistic regression with descriptive statistics and used a Chi-square test to investigate possible cloning patterns. Fourth, we performed an intersectional analysis to investigate whether particular groups had different odds of being a medical specialist than others. Analyses were performed using IBM SPSS software for Windows, Version 25.0 (IBM Corp, Armonk, NY).

### Role of the funding source

The funders had no role in the study design; in the collection, analysis, and interpretation of data; in the writing of the report; and in the decision to submit the paper for publication.

## Results

**Fig. 1:**
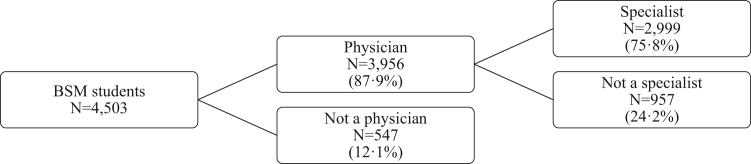
Participant flow diagram.

Table 2 summarizes the demographics of the main populations in this study. A participant flow diagram can be found in Fig. 1. The univariable logistic regression analyses (Appendix 2) show that the variable associated with becoming a physician or (particular type of) medical specialist most often was migration background, which was significantly associated with the outcome in 5 of 5 analyses. This was followed by sex, urbanity degree, and income percentile (all 3/5), and having healthcare professional parents and assets percentile (both 2/5). We found no evidence of multicollinearity in the data. The highest Pearson correlation was 0.339 (p < 0.001), suggesting a weak correlation.^25^ The VIF values varied between 1.010 and 1.198, which are all close to the smallest possible value of VIF (1.0), suggesting an absence of multicollinearity.^26^ All independent variables were included in multivariable regression models performed on the BSM cohort (Appendix 3). In this section, we provide the main significant findings and the corresponding odds ratios (OR) and 95% confidence intervals (CI).

**Table 2:** Demographic characteristics of BSM cohort, physicians and specialists.

Demographic characteristics	1: BSM cohort (N = 4503)	2: Physicians from BSM cohort (N = 3956)	3: Specialists from BSM cohort (N = 2999)	4: CSG Cluster 1 from BSM (N = 1211)	5: CSG Cluster 2 from BSM (N = 1729)	6: CSG Cluster 3 from BSM (N = 59)	7: All physicians (N = 76,845)	8: All specialists of Cluster 1, 2 and 3 combined (N = 49,956)	9: All specialists Cluster 1 (N = 18,630)	10: All specialists Cluster 2 (N = 27,359)	11: All specialists Cluster 3 (N = 3967)
	Frequency (%)	Frequency (%)	Frequency (%)	Frequency (%)	Frequency (%)	Frequency (%)	Frequency (%)	Frequency (%)	Frequency (%)	Frequency (%)	Frequency (%)
Sex											
Male	1498 (33.3)	1250 (31.6)	945 (31.5)	276 (22.8)	652 (37.7)	17 (28.8)	33,092 (43.1)	24,102 (48.2)	7440 (39.9)	14,477 (52.9)	2185 (55.1)
Female	2999 (66.6)	2706 (68.4)	2054 (68.5)	935 (77.2)	1077 (62.3)	42 (71.2)	43,751 (56.9)	25,853 (51.8)	11,189 (60.1)	12,882 (47.1)	1782 (44.9)
Unknown	6 (0.1)						2 (0)	1 (0)	1 (0)		
Migration background											
No migration background	3438 (76.3)	3047 (77)	2408 (80.3)	981 (81)	1390 (80.4)	37 (62.7)	59,326 (77.2)	39,451 (79.0)	15,498 (83.2)	20,878 (76.3)	3075 (77.5)
Turkish or Moroccan MB	95 (2.1)	83 (2.1)	57 (1.9)	24 (2)	30 (1.7)	<10^a^	1233 (1.6)	596 (1.2)	235 (1.3)	326 (1.2)	35 (0.9)
SDI MB	262 (5.8)	220 (5.6)	153 (5.1)	47 (3.9)	98 (5.7)	<10^a^	3495 (4.5)	2070 (4.1)	597 (3.2)	1217 (4.4)	256 (6.5)
European MB	168 (3.7)	150 (3.8)	97 (3.2)	30 (2.5)	64 (3.7)	<10^a^	4836 (6.3)	3083 (6.2)	685 (3.7)	2212 (8.1)	186 (4.7)
Other	540 (12)	456 (11.5)	284 (9.5)	129 (10.7)	147 (8.5)	<10^a^	7955 (10.4)	4756 (9.5)	1615 (8.7)	2726 (10)	415 (10.5)
Urbanity degree^b^											
Very strongly urban	603 (13.4)	520 (13.1)	379 (12.6)	143 (11.8)	223 (12.9)	13 (22.0)	b				
Strongly urban	945 (21.0)	828 (20.9)	624 (20.8)	243 (20.1)	367 (21.2)	14 (23.7)					
Averagely urban	930 (20.7)	812 (20.5)	617 (20.6)	245 (20.2)	362 (20.9)	10 (16.9)					
Little urban	898 (19.9)	806 (20.4)	656 (21.9)	259 (21.4)	383 (22.2)	14 (23.7)					
Not urban	744 (16.5)	665 (16.8)	514 (17.1)	214 (17.7)	296 (17.1)	<10^a^					
Unknown	383 (8.5)	325 (8.2)	209 (7.0)	107 (8.8)	98 (5.7)	<10^a^					
Parental income category											
Percentile 1–70	670 (14.9)	581 (14.7)	422 (14.1)	163 (13.5)	245 (14.2)	14 (23.7)	16,880 (22.0)	12,779 (25.6)	4853 (26.0)	6464 (23.6)	1462 (36.9)
Percentile 71–90	1129 (25.1)	988 (25)	782 (26.1)	359 (29.6)	411 (23.8)	12 (20.3)	17,347 (22.6)	11,371 (22.8)	4468 (24.0)	6161 (22.5)	742 (18.7)
Percentile 91–100	2307 (51.2)	2056 (52)	1600 (53.4)	586 (48.4)	987 (57.1)	27 (45.8)	29,723 (38.7)	16,051 (32.1)	6091 (32.7)	9413 (34.4)	547 (13.8)
Unknown	397 (8.8)	331 (8.4)	195 (6.5)	103 (8.5)	86 (5)		12,895 (16.8)	9755 (19.5)	3218 (17.3)	5321 (19.4)	1216 (30.7)
Parental assets category											
Percentile 1–40	424 (9.4)	356 (9.0)	262 (8.7)	108 (8.9)	148 (8.6)	<10^a^	5617 (7.3)	3080 (6.2)	1153 (6.2)	1637 (6.0)	290 (7.3)
Percentile 41–80	1468 (32.6)	1277 (32.3)	963 (32.1)	386 (31.9)	561 (32.4)	16 (27.1)	19,352 (25.2)	11,304 (22.6)	4510 (24.2)	5927 (21.7)	867 (21.9)
Percentile 81–100	2199 (48.8)	1972 (49.8)	1565 (52.2)	605 (50.0)	931 (53.8)	29 (49.2)	32,260 (42.0)	21,847 (43.7)	8248 (44.3)	12,229 (44.7)	1370 (34.5)
Unknown	412 (9.1)	351 (8.9)	209 (7.0)	112 (9.2)	89 (5.1)	<10^a^	19,616 (25.5)	13,725 (27.5)	4719 (25.3)	7566 (27.7)	1440 (36.3)
Number of HP parents											
No HP parents	3235 (71.8)	2830 (71.5)	2108 (70.3)	875 (72.3)	1184 (68.5)	49 (83.1)	60,912 (79.3)	40,828 (81.7)	15,106 (81.1)	22,043 (80.6)	3679 (92.7)
1 or 2 HP parents	1268 (28.2)	1126 (28.5)	891 (29.7)	336 (27.7)	545 (31.5)	10 (16.9)	15,933 (20.7)	9128 (18.3)	3524 (18.9)	5316 (19.4)	288 (7.3)

Closed specialties are deleted (Appendix 1).

a Data blinded due to CBS regulations, which in general does not allow publication of frequencies <10. Only in rare cases, exceptions are allowed.

b Urbanity degree of postal code area during secondary school could not be calculated for practicing physicians/specialists.

### Demographic characteristics of students associated with becoming a registered physician

Univariable regressions show that female sex (OR 1.83 [1.53–2.20], p < 0.001), having parents in the top-20 assets percentiles (OR 1.66 [1.24–2.23], p = 0.001), and coming from a weakly/not urban postal code area (OR 1.28 [1.03–1.59], p = 0.028), were associated with higher odds of becoming a physician. Having a Turkish, Moroccan, Surinamese, Dutch Caribbean or Indonesian migration background (OR 0.72 [0.53–0.98], p = 0.037) or Other migration background (OR 0.76 [0.60–0.96], p = 0.023) was associated with lower odds. However, in the multivariable regression models, only female students had higher odds to become a physician (OR 1.87 [1.53–2.28], p < 0.001).

### Demographic characteristics of physicians associated with becoming a (particular type of) specialist

The univariable results show that for physicians, several characteristics were associated with higher odds of becoming a specialist: having parents in the top-30 income percentiles (P71–90: OR 1.43 [1.13–1.82], p = 0.003; P91–100: OR 1.32 [1.07–1.63] p = 0.009); having parents in the top-20 assets category (OR 1.38 [1.06–1.79], p = 0.015); coming from a weakly/not urban postal code area (OR 1.34 [1.12–1.59], p = 0.001); and having 1 or 2 healthcare professional parents (OR 1.30 [1.10–1.53], p = 0.002). Having a Turkish, Moroccan, Surinamese, Dutch Caribbean or Indonesian migration background (OR 0.60 [0.46–0.78], p < 0.001) or Other migration background (OR 0.45 [0.37–0.54], p < 0.001) was associated with lower odds. For other univariable results, we refer the reader to Appendix 2 for sake of readability.

The multivariable analyses summarized in Table 3 show that migration background was the only variable significantly associated with becoming a specialist. Physicians with an Other migration background had lower odds of becoming a specialist (OR 0.55 [0.43–0.71], p < 0.001). This was not significant for the group with a Turkish, Moroccan, Surinamese, Dutch Caribbean or Indonesian migration background (OR 0.74 [0.54–1.03], p = 0.073). Female physicians, physicians with parents in the income category 71–90 and physicians without a migration background had higher odds of becoming a specialist in Cluster 1. Men had higher odds of becoming a specialist in Cluster 2. The model of Cluster 3 contained no significant variables. For other multivariable results, we refer the reader to Appendix 3 for sake of readability.

**Table 3:** Summary of cloning pattern analysis.

Specialty	Characteristics of physicians of the BSM cohort associated with significantly higher odds of entering a specialty (cluster)^a^						Cloning pattern, compared to the group average of all physicians/specialists in The Netherlands^b^	Cloning pattern, compared to physicians without a specialty or compared to the other clusters, incl. p-value^c^	Supportive of reproduction of sameness?
	Sex	Migration background	Income percentile	Assets percentile	Nr. of HP parents	Urbanity degree			
Any specialty		No migration background					Specialists in the Netherlands more often have no migration background (79.0%), compared to all physicians (77.2%)	Specialists in the Netherlands significantly more often have no migration background (79.0%), compared to physicians without a specialty (73.9%) [p < 0.00001]	Yes
Cluster 1 (Family medicine; elderly care medicine; intellectual disability medicine)	Female	No migration background	71–90				Specialists in Cluster 1 are more often female (60.1%), compared to all specialists combined (51.8%). Specialists in Cluster 1 more often have no migration background (83.2%) compared to all specialists combined (79.0%). Specialists in Cluster 1 more often have parents in the 71–90 income category (24.0%) compared to all specialists combined (22.8%).	Specialists in Cluster 1 are significantly more often female (60.1%), compared to specialists in Cluster 2 & 3 (46.8%) [p < 0.00001]. Specialists in Cluster 1 significantly more often have no migration background (83.2%) compared to specialists in Clusters 2 & 3 (76.4%) [p < 0.00001]. When missings are not excluded, specialists in Cluster 1 significantly more often have parents in the 71–90 income category (24.0%) compared to specialists in Clusters 2 & 3 (22.0%) [p < 0.00001]. When missings are excluded, specialists in Cluster 1 significantly more often have parents in the 71–90 income category (29.0%) compared to specialists in Clusters 2 & 3 (27.8%) [p = 0.013,331].	Yes
Cluster 2 (all hospital specialties)	Male						Specialists in Cluster 2 are more often male (52.9%), compared to all specialists combined (48.2%).	Specialists in Cluster 2 are significantly more often male (52.9%), compared to specialists in Clusters 1 & 3 (42.5%) [p < 0.00001].	Yes
Cluster 3 (occupational medicine; insurance medicine; public health medicine)							No significant variables in the multivariable model.	No significant variables in the multivariable model.	Not applicable

a Each row shows which category within a variable was associated with significantly (p < 0.05) higher odds of entering a specialty or group of specialties, according to the multivariable logistic regression models in Appendix 3. Empty cells indicate that the variable was not significantly associated with the outcome.

b Cloning pattern compared to the group average of all physicians/specialists: we compared the demographic characteristics of specialty clusters (in Table 2) in with the demographics of the entire physician population of The Netherlands (Table 2, column 7). We focused on the demographic characteristics which resulted in significantly higher odds of entering the specialty (summarized in columns 2–7 of this table).

c Cloning pattern, compared to the other clusters: We analyzed whether the demographics mentioned in columns 2–7 of this table were significantly different from the comparison group, using the Chi-square test of association with p < 0.05.

### Cloning pattern of the existing workforce

We investigated whether the demographic characteristics of the physicians from the BSM cohort which resulted in higher odds were already overrepresented in that specific specialist workforce on a national level. The results are summarized in the last three columns of Table 3, which describes whether the multivariable models in Appendix 3 are supportive of a cloning pattern or not. We compared the percentages of a given variable category to the total specialist population’s demographics; and to the demographics of the other clusters. In all analyses except Cluster 3 (which had no significant variables in the model), a cloning pattern was observed. The Chi-square test of association showed that clusters were significantly different from each other on the basis of the compared variables. The largest differences were found in relation to sex and migration background. While parental income category was statistically significant for Cluster 1, its practical relevance is less substantial as, when missings are excluded, the difference with Clusters 2 & 3 is small (1.2 percentage points). Therefore, this section focuses only on sex and migration background.

**Fig. 2:**
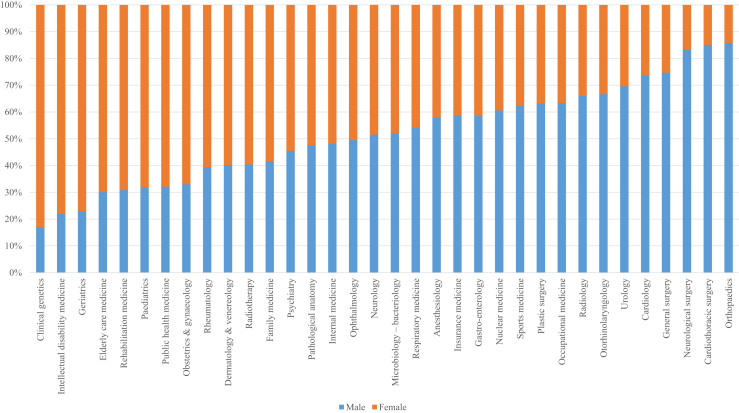
Sex distribution within all medical specialties in the Netherlands in 2021.

Male physicians from the BSM cohort had higher odds of specializing in Cluster 2, and specialists in Cluster 2 are significantly more often male than in Clusters 1 & 3 combined. However, there are large differences within Cluster 2 on a national level with regard to their male/female distribution (see Appendix 4 for descriptive statistics per specialty). For example, 85.7% of orthopaedic surgeons are men, while 83.1% of clinical geneticists are women. These substantial differences are visualized in Fig. 2.

**Fig. 3:**
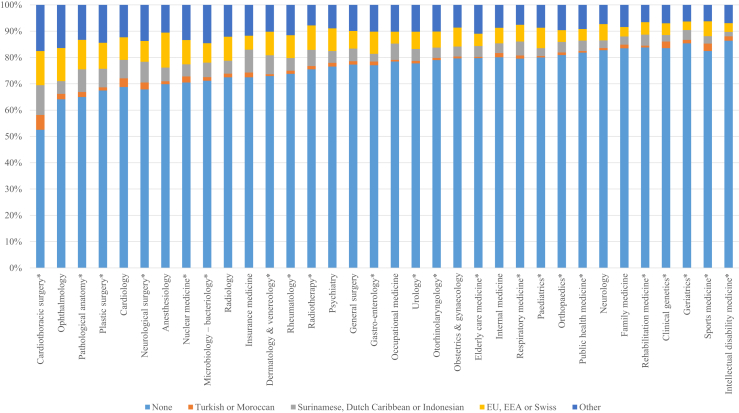
Migration backgrounds within all medical specialties in the Netherlands in 2021.

Physicians with a migration background had lower odds of specialization. However, there are a few specialties in which they are well represented on a national level. Examples include cardiothoracic surgery (44.6%), pathological anatomy (34.0%) and plastic surgery (31.6%). Nevertheless, in all three examples, there are fewer than 10 specialists with a Turkish or Moroccan migration background. This was also the case for the fields with the lowest proportion of specialists with a migration background: intellectual disability medicine (11.8%), sports medicine (12.6%) and geriatrics (13.3%). The differences between specialties are summarized in Fig. 3.

*∗ specialties marked with an asterisk had either <5 or <10 specialists with a Turkish or Moroccan migration background. Due to Statistics Netherlands regulations, exact percentages are not allowed to be shared for these cases. We therefore calculated percentages as if there were exactly 5 or 10 specialists with these backgrounds, and deducted the difference from the group ‘No migration background’. This results in small rounding errors, slightly distorting the blue bars for ‘None’*.

If both sex and migration background are considered simultaneously, none of the specialties’ demographics in Appendix 4 currently reflect the demographics of the BSM cohort. This led us to further investigate a possible cloning pattern. We performed an intersectional analysis, combining sex and migration background, resulting in ten categories. The left column in Table 4 (based on the national pool of physicians) shows that compared to men without a migration background, every other group had lower odds of being a specialist. The difference was the smallest for men with an European migration background. Female physicians with a Turkish or Moroccan migration background had the lowest odds of all, followed by women with an Other migration background and women with a Surinamese, Dutch Caribbean or Indonesian migration background. When we performed the same analysis on the BSM cohort, the lower odds of specializing compared to men without a migration background were only significant for women with a migration background other than Turkish or Moroccan, and for men with an Other migration background. Appendix 5 contains the descriptive statistics of these intersectional groups. A comparison of both tables in Appendix 5 indicates that 5 out of 10 groups of physicians showed (slightly) improved representation amongst specialists in the BSM cohort, compared to the total population of specialists: women without a migration background, men and women with a Turkish/Moroccan background; women with a Surinamese/Dutch Caribbean/Indonesian background and women with Other (non-European) backgrounds.

**Table 4:** Results from the intersectional analysis, combining sex and migration background.

	From NL physician to NL specialist	p value	From BSM physician to specialist	p value
	Unadjusted OR (95% C.I.)		Unadjusted OR (95% C.I.)	
Women without migration background (ref. MW)	0.53 (0.51–0.55)	<0.001	0.98 (0.81–1.19)	0.82
Men with Turkish/Moroccan MB (ref. MW)	0.43 (0.36–0.51)	<0.001	0.50 (0.25–1.00)	0.05
Women with Turkish/Moroccan MB (ref. MW)	0.26 (0.22–0.30)	<0.001	0.64 (0.33–1.30)	0.19
Men with Surinamese/Dutch Caribbean/Indonesian MB (ref. MW)	0.71 (0.64–0.79)	<0.001	0.71 (0.41–1.21)	0.20
Women with Surinamese/Dutch Caribbean/Indonesian MB (ref. MW)	0.38 (0.35–0.42)	<0.001	0.55 (0.38–0.81)	<0.001
Men with European MB (ref. MW)	0.84 (0.77–0.94)	0.001	0.78 (0.41–1.50)	0.46
Women with European MB (ref. MW)	0.47 (0.43–0.51)	<0.001	0.38 (0.25–0.59)	<0.001
Men with other MB (ref. MW)	0.73 (0.68–0.79)	<0.001	0.41 (0.29–0.58)	<0.001
Women with other MB (ref. MW)	0.38 (0.35–0.40)	<0.001	0.45 (0.33–0.60)	<0.001

MW: men without migration background (reference category).

## Discussion

Our results suggest that the medical specialist population is not representative of the medical student population and the physician population. The loss of diversity in the route to becoming a specialist starts early in life, impacting medical school enrollment.^20^ The student body is predominantly female and from a high SES background, and students with certain migration backgrounds are underrepresented. Male students are less likely to become a physician. The lower odds of specialization for physicians with a migration background contribute to their underrepresentation, especially when they are also female.

We do not know why male students are less likely to become a physician. The OECD has reported that across the world, male students have lower levels of college completion than female students.^27^ The leaky pipeline for men up until graduation of medical school has not yet resulted in the underrepresentation of men in the *specialist* workforce. In only 8 out of 34 specialties, they make up less than 35% of the workforce, while having made up less than 35% of medical students for over 20 years. In 18 specialties, men are still in the majority. This may partially be due to historical reasons: until 1989, men constituted more than 50% of the medical student population. In 2001, for the first time, half of all residents in training were female.^28^ The high odds of specialization for men were also supported by the intersectional regression analyses performed on the national pool of physicians. These results showed that the four groups with the highest odds of specialization were all men: men without a migration background, men with a European background, men with an Other background, and men with a Surinamese, Dutch Caribbean or Indonesian background. Only men with a Turkish or Moroccan background had lower odds of specialization than the groups of women with the highest odds (those without a migration background and with a European background).

Essed’s theory on cultural cloning was supported in all analyses except Cluster 3, which did not have significant variables in its model. This is likely due to its small size: only 59 BSM students became a specialist in Cluster 3. The significant variables in the other analyses were sex and migration background. This means that physicians are more likely to specialize in a cluster where people of their sex and/or migration background are overrepresented. The intersectional analyses showing the highest odds of specialization, on a national level, for male physicians without a migration background or with a European background. The lowest odds were found for women who, based on their countries of origin, are likely to be women of color. These findings are supportive of Essed’s claim that

*“the cultural process of ‘cloning the physician’ is facilitated in at least the following two ways: (a) through the dominance of masculine, European norms and values in medical cultures, and (b) by relating interpretations of medical competence to these norms and values. There is also an embodied dimension to cultural cloning: the white male body speaks to perceivers’ sense of whiteness, and (Dutch) masculinity. Therefore, (middle class) white men will be attributed more generously than others traits qualifying them as medically competent. Others (non-whites, non-males) may have to prove more vigorously that they master the qualifying criteria in spite of their different bodies”* (p. 132).

This study is the first to confirm the previously suggested pattern that in The Netherlands, doctors with a migration background are less likely to become a specialist,^7^ aligning with research showing lower hiring rates for residency training applicants with a migration background.^4^ However, the previous estimate that 2–4% of hospital specialists have a migration background^21^ requires nuancing: in 2021, there were 6481 specialists with a migration background in Cluster 2 (23.7% of hospital specialists). While this may seem in line with the general population, where 25.2% had a migration background in 2022,^22^ certain groups within the workforce remain sharply underrepresented compared to the society. For example, Turkish-Dutch and Moroccan-Dutch citizens (of the 1st and 2nd generation) comprise 4.8% of the population,^29^ but constitute 1.6% of physicians and 1.2% of the specialist workforce. In every specialty, they were underrepresented. This underrepresentation amongst physicians by a factor 3 (4.8/1.6) and specialists by a factor 4 (4.8/1.2) is larger than the underrepresentation of e.g., Black (factor 2.76) or Hispanic physicians (factor 2.88) in the United States.^30^ Other groups are also underrepresented compared to the ethnic diversity of the society. This can negatively impact clinical practice.^24^ For example, studies show that increased diversity amongst healthcare professionals can result in improved patient satisfaction, improved therapy compliance, improved communication and patient trust, and improved medical research on ‘minority diseases’.^1^^,^^6^^,^^10^^,^^31^ There are benefits to when patient and healthcare provider share the same ethnic background. For example, Black patients in the US have better life expectancy in counties where there is greater representation of Black primary care physicians.^32^ Witnessing the lack of diversity of skin tones in medical resources, Black medical students (e.g., Malone Mukwende), illustrators (e.g., Chidiebere Ibe) and dermatologists published resources focused on skin conditions on black and brown skin, thereby improving healthcare for a significant part of the world’s population.^33^ Next to patients, healthcare providers who belong to underrepresented groups in medicine themselves also experience harmful effects of the lack of representation. Examples include isolation, discrimination, racism, feeling a lack of belonging, experiencing ‘othering’, unfair treatment, exclusion, feeling hyper-visible, and other types of inequality at all stages of their education and career trajectory.^34^

Due to the absence of a national application system in The Netherlands for residency training positions, we do not know to what extent our results can be explained by self-selection or inequality in the hiring procedures. This warrants future research on why these groups are underrepresented, e.g., by investigating the recruitment and hiring procedures, and on how the healthcare sector can systematically empower them. It is possible that certain groups (perceive to) have lower chances of being hired in competitive specialties, leading them to apply for less competitive specialties, or that they have different preferences. For example, UK research shows that sex-based segregation in specialties can be attributed to different application rates, not bias in the instruments used for selection.^35^ If this is the case in The Netherlands, it would provide grounds to aim improved recruitment efforts targeted to underrepresented populations. At the same time, studies indicate that the hiring processes for medical personnel, including for residency training positions, can be non-transparent^18^ and result in an adverse impact for certain groups of applicants.^3^^,^^4^^,^^19^ This can be due to (similarity) bias,^5^^,^^7^^,^^18^ thereby (unconsciously) excluding suitable applicants from underrepresented backgrounds.^18^ Implicit norms of medical professionalism and performance, partly based on stereotyped ways of thinking, and by (unintended) exclusionary practices in the workplace, have also been reported.^7^

### Strengths, limitations and future research

A strength of the study is that we used a retrospective cohort design in which we were able to combine data from the national register of all physicians and specialists with an active registration with a diverse range of background characteristics as registered by Statistics Netherlands. Through the combination of quantitative statistical analyses with Essed’s theory of cultural cloning, we have been able to shed light on intersectional elements of the leaky pipeline in medicine in a scientifically rigorous and original manner. Our approach can be replicated in other (international) contexts as well, making it a valuable addition to the academic literature. Especially in contexts where detailed demographic data on sex, migration background, ethnicity and other variables are available, future research using intersectional analyses can shed further light on the domains (both within and outside medicine) in which the workforce composition may support or contradict Essed’s theory of cultural cloning of e.g., whiteness and masculinity.

This study has several limitations, affecting the generalizability which is limited to our setting. First, our data are historical. They show what happened between 2002 and 2021, and the regression models cannot be used as prediction models. The diversity of the BSM cohort may not reflect the diversity of current incoming medical students. Second, an active BIG registration does not mean someone is *employed* as a physician/specialist, only that someone is *allowed* to be employed as such. Future research could investigate actual employment, to find out how representative the workfloors within the healthcare sector are in reality. Third, we investigated whether someone became a registered physician/specialist, but not how long it took them to achieve their registration. Analyzing possible time-to-event differences using Kaplan–Meier survival analysis would enable a deeper understanding of specific barriers that may exist for specific groups within the leaky pipeline. Future studies could analyze differences in time-to-event duration, as this may be an element of the leaky pipeline which was outside the scope of this present study. To enable such an analysis, it would be necessary to know the dates on which physicians started their specialty training. This is currently not a part of Statistics Netherlands datasets, which was a fourth limitation that made it impossible to determine which physicians were currently in specialty training. Fifth, background characteristics such as (in)visible disabilities, sexual orientation, gender identity, skin color, or religion may influence physicians’ career opportunities and choices, but such data are not available. However, visible role models are important in medicine.^36^^,^^37^ Future research could investigate the representativeness of the medical workforce with regard to these characteristics on a larger scale, building upon e.g., Tweed et al.^38^

### Recommendations

In light of the importance of inclusive healthcare systems, we recommend that every recruitment body increases the representativeness of their workforce. Implementation of a national registration system for applications and selection outcomes for residency training would promote transparency, and would enable investigation of potential self-selection and bias. While allowing local and regional hiring bodies to select their residents in training, a national registration system is necessary to find out what causes the loss of diversity in the step from physician to (a particular type of) specialist. Only with these data can be determined to what extent certain groups apply in higher/lower rates to particular specialties than others, and to what extent there is inequality in the selection procedures for residency training. To enable a deeper investigation of different elements of the leaky pipeline, we also recommend that certain data of physicians who are a enrolled in specialty training become a part of Statistics Netherlands datasets.

Specialists with a Turkish or Moroccan background (men and women) and women with a non-European migration background are the least likely to be a specialist. Addressing the systematic issue of representativeness requires careful consideration of various solutions. While increasing underrepresented members within the hiring committee can be helpful, it may not suffice in overcoming the dearth of medical specialists with a migration background. An alternative approach could be to adopt preferential hiring practices for residency training and specialist positions, which necessitates a holistic and contextualized evaluation of an individual's merit and assets beyond their performance.^18^^,^^39^ This would require acknowledging the socio-economic challenges, racism, and power imbalances that impede the progress of underrepresented groups,^40^ as well as recognizing the potential contribution of these healthcare professionals towards creating an equitable healthcare system.^8^ To achieve this, efforts should start before *and* at the medical school admissions stage, with the aim of increasing the pool of potential residency applicants from underrepresented backgrounds.^9^

## Contributors

LM conceived the study. All authors contributed to the evolution of the study conceptualization. As data is owned and anonymized by Statistics Netherlands, data curation and validation did not apply. Software was provided by Statistics Netherlands. LM, MS, SP and AW developed the initial methodology, which was refined by EUA, ASK and LM. LM and RAK performed the literature search. LM gained access to the data, scrubbed the data, performed all formal analyses, visualized results, wrote the original draft and was the project administrator. All authors contributed to the interpretation of the results, and to reviewing and editing subsequent versions of the manuscript. RAK procured funding and resources to carry out the study. AW, RAK, GC and JHR supervised the study.

## Data sharing statement

All original microdata are non-public, anonymized and owned by Statistics Netherlands. Under certain conditions, these microdata are accessible for statistical and scientific research. For further information: microdata@cbs.nl. This study could be replicated if access to the datasets is gained. Our study protocol, including all procedures, sources of variables, statistical analysis plan, and software syntax for statistical analysis, can be found in the Supplementary Material.

## Ethical considerations

The Ethics Committee at Amsterdam UMC approved the study (file no. 2022.0540). The statistical results are based on calculations by Lianne Mulder (Amsterdam UMC) using non-public anonymized microdata from Statistics Netherlands, and comply to all Statistics Netherlands privacy regulations and the Dutch law regarding use of their non-public microdata (Wet op het Centraal Bureau voor de Statistiek, 2004). The researchers had no access to identifiable information.

## Declaration of interests

The authors declare that they have no competing interests.

## Appendix A Supplementary data

Appendices 1–5

Research protocol
