# Supplementary material for: Diversity in the pathway from medical student to specialist in the Netherlands: a retrospective cohort study
Source: Lancet Reg Health Eur. 2023 Oct 12;35:100749. doi: 10.1016/j.lanepe.2023.100749 (PMC10583163; doi:10.1016/j.lanepe.2023.100749)
Supplement: Appendices 1–5 [file mmc1.docx]

# **Appendix 1: Clusters of specialties**

| **Cluster 1** |  |  |
| --- | --- | --- |
| General medical practice/family medicine | Elderly care medicine | Intellectual disability medicine |
|  |  |  |
| **Cluster 2 (hospital based specialties)** |  |  |
| Anaesthesiology | Respiratory medicine | Pathological anatomy |
| Cardiology | Gastroenterology | Plastic surgery |
| Cardiothoracic surgery | Microbiology-bacteriology | Psychiatry |
| Dermato-venereology | Neurological surgery | Diagnostic radiology |
| General surgery | Neurology | Radiotherapy |
| General (internal) medicine | Nuclear medicine | Rheumatology |
| Otorhinolaryngology | Obstetrics and gynaecology | Rehabilitation medicine |
| Paediatrics | Ophthalmology | Sports medicine |
| Clinical genetics | Orthopaedics | Urology |
| Geriatrics |  |  |
|  |  |  |
| **Cluster 3** |  |  |
| Occupational medicine – domain labour and health | Insurance medicine – domain labour and health | Public health medicine |

Note to the international reader: In The Netherlands, the following areas of medical specialization are defined as profiles within Cluster 1, 2 and 3. They are not recognized as official specialties by the Royal Association of Medicine (KNMG), and are therefore not included in the dataset as they are not registered in the BIG register:

**Cluster 1 profiles**

Global health and tropical medicine

Addiction medicine

Cosmetic medicine

**Cluster 2 profiles**

Accident and emergency medicine

Hospital medicine

**Cluster 3 profiles**

Policy and advisory medicine

Donor medicine

Forensic medicine

Communicable disease control

Youth health care

Medical environmental science

Social-medical assessment and advisory medicine

Tuberculosis control

The specialties of Allergology and Biological chemistry are closed: physicians can no longer register in these specialties. Therefore, they are excluded from this study.

# **Appendix 2: Results from the univariable logistic regression analyses performed on the BSM cohort**

Due to Statistics Netherlands regulations, some results are hidden, as they are based on frequencies <10. Statistics Netherlands requires removal of these results to avoid traceability to an individual or small groups of individuals. We replaced the content of the regression result with ‘Hidden’.

|  | **FROM BSM TO PHYSICIAN** |  | **FROM PHYSICIAN TO SPECIALIST** |  |
| --- | --- | --- | --- | --- |
|  | Unadjusted OR (95% CI) | p value | Unadjusted OR (95% CI) | p value |
| **SEX** |  |  |  |  |
| Female (ref. Male) | 1·83 (1·53-2·20) | **<0·001** | 1·02 (0·87-1·19) | 0·835 |
| **MIGRATION BACKGROUND** |  |  |  |  |
| TMSDI (ref. none) | 0·72 (0·53-0·98) | **0·037** | 0·60 (0·46-0·78) | **<0·001** |
| European or Other (ref. none) | 0·76 (0·60-0·96) | **0·023** | 0·45 (0·37-0·54) | **<0·001** |
| **URBANITY DEGREE OF POSTAL CODE DURING ADOLESCENCE** |  |  |  |  |
| Averagely urban (ref. (very) strongly urban) | 1·02 (0·80-1·30) | 0·867 | 1·09 (0·89-1·33) | 0·412 |
| Weakly to not urban (ref. very strongly urban) | 1·28 (1·03-1·59) | **0·028** | 1·34 (1·12-1·59) | **0·001** |
| **INCOME PERCENTILE** |  |  |  |  |
| Percentile 71-90 (ref. 1-70) | 1·07 (0·81-1·43) | 0·626 | 1·43 (1·13-1·82) | **0·003** |
| Percentile 91-100 (ref. 1-70) | 1·26 (0·97-1·63) | 0·086 | 1·32 (1·07-1·63) | **0·009** |
| **ASSETS PERCENTILE** |  |  |  |  |
| Percentile 41-80 (ref. 1-40) | 1·28 (0·95-1·73) | 0·111 | 1·10 (0·84-1·44) | 0·484 |
| Percentile 81-100 (ref. 1-40) | 1·66 (1·24-2·23) | **0·001** | 1·38 (1·06-1·79) | **0·015** |
| **NUMBER OF HEALTHCARE PROFESSIONAL PARENTS** |  |  |  |  |
| 1 or 2 parents (ref. 0 parents) | 1·14 (0·93-1·39) | 0·223 | 1·30 (1·10-1·53) | **0·002** |

OR: odds ratio; CI: confidence interval; TMSDI: Turkish, Moroccan, Surinamese, Dutch Caribbean or Indonesian migration background

|  | **FROM PHYSICIAN TO CLUSTER 1** |  | **FROM PHYSICIAN TO CLUSTER 2** |  | **FROM PHYSICIAN TO CLUSTER 3** |  |
| --- | --- | --- | --- | --- | --- | --- |
|  | Unadjusted OR (95% CI) | p value | Unadjusted OR (95% CI) | p value | Unadjusted OR (95% CI) | p value |
| **SEX** |  |  |  |  |  |  |
| Female (ref. Male) | 1·86 (1·60-2·18) | **<0·001** | 0·61 (0·53-0·69) | **<0·001** | 1·14 (0·65-2·02) | 0·643 |
| **MIGRATION BACKGROUND** |  |  |  |  |  |  |
| TMSDI (ref. none) | 0·64 (0·49-0·85) | **0·002** | 0·87 (0·69-1·11) | 0·261 | Hidden | **0·001** |
| European or Other (ref. none) | 0·75 (0·62-0·91) | **0·004** | 0·64 (0·53-0·76) | **<0·001** | Hidden | 0·239 |
| **URBANITY DEGREE OF POSTAL CODE DURING ADOLESCENCE** |  |  |  |  |  |  |
| Averagely urban (ref. (very) strongly urban) | 1·08 (0·89-1·30) | 0·447 | 1·03 (0·87-1·23) | 0·712 | 0·61 (0·29-1·27) | 0·185 |
| Weakly to not urban (ref. very strongly urban) | 1·18 (1·01-1·39) | **0·043** | 1·10 (0·95-1·28) | 0·203 | 0·61 (0·33-1·11) | 0·103 |
| **INCOME PERCENTILE** |  |  |  |  |  |  |
| Percentile 71-90 (ref. 1-70) | 1·46 (1·17-1·83) | **0·001** | 0·98 (0·79-1·20) | 0·825 | 0·50 (0·23-1·08) | 0·079 |
| Percentile 91-100 (ref. 1-70) | 1·02 (0·83-1·26) | 0·833 | 1·27 (1·05-1·53) | **0·013** | 0·54 (0·28-1·04) | 0·063 |
| **ASSETS PERCENTILE** |  |  |  |  |  |  |
| Percentile 41-80 (ref. 1-40) | 1·00 (0·77-1·29) | 0·968 | 1·10 (0·87-1·40) | 0·427 | Hidden | 0·533 |
| Percentile 81-100 (ref. 1-40) | 1·02 (0·80-1·30) | 0·897 | 1·26 (1·00-1·58) | 0·05 | Hidden | 0·759 |
| **NUMBER OF HEALTHCARE PROFESSIONAL PARENTS** |  |  |  |  |  |  |
| 1 or 2 parents (ref. 0 parents) | 0·95 (0·82-1·11) | 0·507 | 1·30 (1·14-1·50) | **<0·001** | 0·51 (0·26-1·01) | 0·053 |

OR: odds ratio; CI: confidence interval; TMSDI: Turkish, Moroccan, Surinamese, Dutch Caribbean or Indonesian migration background

# **Appendix 3: Results from the multivariable logistic regression analyses**

Due to Statistics Netherlands regulations, some results are blinded, as they are based on frequencies <10. Statistics Netherlands requires removal of these results to avoid traceability to an individual or small groups of individuals. We replaced the content of the regression result with ‘Hidden’.

|  | **FROM BSM TO PHYSICIAN** |  | **FROM BSM PHYSICIAN TO SPECIALIST** |  |
| --- | --- | --- | --- | --- |
|  | **Adjusted OR (95%CI)** | **p value** | **Adjusted OR (95%CI)** | **p value** |
| **SEX** |  |  |  |  |
| Female (ref. Male) | 1·87 (1·53-2·28) | **<0·001** | 0·97 (0·81-1·15) | 0·7 |
| **MIGRATION BACKGROUND** |  |  |  |  |
| TMSDI (ref. none) | 0·83 (0·57-1·20) | 0·317 | 0·74 (0·54-1·03) | 0·073 |
| European or Other (ref. none) | 1·02 (0·74-1·42) | 0·903 | 0·55 (0·43-0·71) | **<0·001** |
| **URBANITY DEGREE OF POSTAL CODE DURING ADOLESCENCE** |  |  |  |  |
| Averagely urban (ref. (very) strongly urban) | 0·94 (0·73-1·21) | 0·627 | 0·99 (0·80-1·22) | 0·921 |
| Weakly to not urban (ref. very strongly urban) | 1·18 (0·94-1·49) | 0·164 | 1·20 (1·00-1·45) | 0·055 |
| **INCOME PERCENTILE** |  |  |  |  |
| Percentile 71-90 (ref. 1-70) | 1·00 (0·74-1·37) | 0·988 | 1·17 (0·90-1·51) | 0·252 |
| Percentile 91-100 (ref. 1-70) | 1·13 (0·84-1·52) | 0·407 | 1·04 (0·81-1·32) | 0·782 |
| **ASSETS PERCENTILE** |  |  |  |  |
| Percentile 41-80 (ref. 1-40) | 1·17 (0·84-1·65) | 0·358 | 0·85 (0·63-1·15) | 0·298 |
| Percentile 81-100 (ref. 1-40) | 1·38 (0·98-1·94) | 0·069 | 1·03 (0·76-1·39) | 0·857 |
| **NUMBER OF HEALTHCARE PROFESSIONAL PARENTS** |  |  |  |  |
| 1 or 2 parents (ref. 0 parents) | 1·06 (0·85-1·33) | 0·607 | 1·13 (0·94-1·36) | 0·198 |

OR: odds ratio; CI: confidence interval; TMSDI: Turkish, Moroccan, Surinamese, Dutch Caribbean or Indonesian

|  | **FROM PHYSICIAN TO CLUSTER 1** |  | **FROM PHYSICIAN TO CLUSTER 2** |  | **FROM PHYSICIAN TO CLUSTER 3** |  |
| --- | --- | --- | --- | --- | --- | --- |
|  | **Adjusted OR (95%CI)** | **p value** | **Adjusted OR (95%CI)** | **p value** | **Adjusted OR (95%CI)** | **p value** |
| **SEX** |  |  |  |  |  |  |
| Female (ref. Male) | 1·91 (1·61-2·26) | **<0·001** | 0·58 (0·50-0·67) | **<0·001** | 1·15 (0·61-2·15) | 0·663 |
| **MIGRATION BACKGROUND** |  |  |  |  |  |  |
| TMSDI (ref. none) | 0·71 (0·51-0·99) | **0·041** | 0·99 (0·74-1·32) | 0·942 | Hidden | 0·064 |
| European or Other (ref. none) | 0·55 (0·41-0·73) | **<0·001** | 0·97 (0·77-1·22) | 0·776 | Hidden | 0·718 |
| **URBANITY DEGREE OF POSTAL CODE DURING ADOLESCENCE** |  |  |  |  |  |  |
| Averagely urban (ref. (very) strongly urban) | 1·04 (0·85-1·27) | 0·705 | 0·99 (0·83-1·19) | 0·913 | 0·58 (0·26-1·30) | 0·186 |
| Weakly to not urban (ref. very strongly urban) | 1·09 (0·92-1·30) | 0·308 | 1·08 (0·93-1·27) | 0·323 | 0·64 (0·34-1·23) | 0·179 |
| **INCOME PERCENTILE** |  |  |  |  |  |  |
| Percentile 71-90 (ref. 1-70) | 1·27 (1·00-1·62) | **0·048** | 0·94 (0·76-1·18) | 0·616 | 0·51 (0·22-1·19) | 0·119 |
| Percentile 91-100 (ref. 1-70) | 0·88 (0·70-1·10) | 0·255 | 1·20 (0·97-1·48) | 0·09 | 0·62 (0·29-1·31) | 0·21 |
| **ASSETS PERCENTILE** |  |  |  |  |  |  |
| Percentile 41-80 (ref. 1-40) | 0·81 (0·61-1·08) | 0·149 | 1·04 (0·80-1·35) | 0·769 | Hidden | 0·493 |
| Percentile 81-100 (ref. 1-40) | 0·86 (0·65-1·13) | 0·28 | 1·11 (0·86-1·44) | 0·423 | Hidden | 0·19 |
| **NUMBER OF HEALTHCARE PROFESSIONAL PARENTS** |  |  |  |  |  |  |
| 1 or 2 parents (ref. 0 parents) | 1·04 (0·88-1·23) | 0·661 | 1·09 (0·94-1·27) | 0·258 | 0·48 (0·23-1·02) | 0·057 |

OR: odds ratio; CI: confidence interval; TMSDI: Turkish, Moroccan, Surinamese, Dutch Caribbean or Indonesian

# **Appendix 4: Descriptive statistics of all specialists with an active BIG registration in the Netherlands**

Due to Statistics Netherlands regulations, some frequencies and percentages are combined or removed, to avoid traceability to an individual or small groups of individuals.

Note: missing data on parental income/assets percentile may partially be influenced by deceased parents of (elderly) specialists

Urbanity degree of postal code at secondary school age could not be determined for all specialists, due to large differences in age and missing data for older specialists.

|  | **FAMILY MEDICINE (N=16260)** | | **PSYCHIATRY (N=3986)** | | **INTERNAL MEDICINE (N=2868)** | | **ANESTHESIOLOGY (N=2188)** | |
| --- | --- | --- | --- | --- | --- | --- | --- | --- |
|  | Frequency | Percentage | Frequency | Percentage | Frequency | Percentage | Frequency | Percentage |
| **SEX** |  |  |  |  |  |  |  |  |
| Male | 6750 | 41·5 | 1814 | 45·5 | 1385 | 48·3 | 1270 | 58·0 |
| Female | 9509 | 58·5 | 2172 | 54·5 | 1483 | 51·7 | 918 | 42·0 |
| Missing | 1 |  |  |  |  |  |  |  |
| **MIGRATION BACKGROUND** |  |  |  |  |  |  |  |  |
| No migration background | 13577 | 83·5 | 3051 | 76·5 | 2296 | 80·1 | 1527 | 69·8 |
| Turkish or Moroccan | 228 | 1·4 | 56 | 1·4 | 47 | 1·6 | 25 | 1·1 |
| Surinamese, Dutch Caribbean or Indonesian | 511 | 3·1 | 179 | 4·5 | 107 | 3·7 | 114 | 5·2 |
| European | 578 | 3·6 | 344 | 8·6 | 168 | 5·9 | 292 | 13·3 |
| Other | 1366 | 8·4 | 356 | 8·9 | 250 | 8·7 | 230 | 10·5 |
| **INCOME PERCENTILE** |  |  |  |  |  |  |  |  |
| Percentile 1-70 | 4122 | 25·4 | 1070 | 26·8 | 724 | 25·2 | 535 | 24·5 |
| Percentile 71-90 | 3904 | 24·0 | 827 | 20·7 | 678 | 23·6 | 451 | 20·6 |
| Percentile 91-100 | 5534 | 34·0 | 1134 | 28·4 | 1009 | 35·2 | 658 | 30·1 |
| Missing | 2700 | 16·6 | 955 | 24·0 | 457 | 15·9 | 544 | 24·9 |
| **ASSETS PERCENTILE** |  |  |  |  |  |  |  |  |
| Percentile 1-40 | 1011 | 6·2 | 268 | 6·7 | 183 | 6·4 | 148 | 6·8 |
| Percentile 41-80 | 3926 | 24·1 | 911 | 22·9 | 644 | 22·5 | 490 | 22·4 |
| Percentile 81-100 | 7297 | 44·9 | 1551 | 38·9 | 1317 | 45·9 | 829 | 37·9 |
| Missing | 4026 | 24·8 | 1256 | 31·5 | 724 | 25·2 | 721 | 33·0 |
| **NUMBER OF HEALTHCARE PROFESSIONAL PARENTS** |  |  |  |  |  |  |  |  |
| 0 parents | 13063 | 80·3 | 3373 | 84·6 | 2308 | 80·5 | 1793 | 81·9 |
| 1 or 2 parents | 3197 | 19·7 | 613 | 15·4 | 560 | 19·5 | 395 | 18·1 |

--

|  | **ELDERLY CARE MEDICINE (N=2073)** | | **OCCUPATIONAL MEDICINE (N=1905)** | | **PAEDIATRICS (N=1830)** | | **GENERAL SURGERY (N=1667)** | |
| --- | --- | --- | --- | --- | --- | --- | --- | --- |
|  | Frequency | Percentage | Frequency | Percentage | Frequency | Percentage | Frequency | Percentage |
| **SEX** |  |  |  |  |  |  |  |  |
| Male | 625 | 30·1 | 1208 | 63·4 | 582 | 31·8 | 1246 | 74·7 |
| Female | 1448 | 69·9 | 697 | 36·6 | 1248 | 68·2 | 421 | 25·3 |
| **MIGRATION BACKGROUND** |  |  |  |  |  |  |  |  |
| No migration background | 1659 | 80·0 | 1496 | 78·5 | 1470 | 80·3 | 1289 | 77·3 |
| Turkish or Moroccan | <10 | blinded | 11 | 0·6 | <10 | blinded | 21 | 1·3 |
| Surinamese, Dutch Caribbean or Indonesian | <85 | blinded | 118 | 6·2 | <55 | blinded | 80 | 4·8 |
| European | 97 | 4·7 | 85 | 4·5 | 143 | 7·8 | 112 | 6·7 |
| Other | 228 | 11·0 | 195 | 10·2 | 159 | 8·7 | 165 | 9·9 |
| **INCOME PERCENTILE** |  |  |  |  |  |  |  |  |
| Percentile 1-70 | 656 | 31·6 | 723 | 38·0 | 386 | 21·1 | 351 | 21·1 |
| Percentile 71-90 | 477 | 23·0 | 355 | 18·6 | 496 | 27·1 | 370 | 22·2 |
| Percentile 91-100 | 473 | 22·8 | 246 | 12·9 | 642 | 35·1 | 669 | 40·1 |
| Missing | 467 | 22·5 | 581 | 30·5 | 306 | 16·7 | 277 | 16·6 |
| **ASSETS PERCENTILE** |  |  |  |  |  |  |  |  |
| Percentile 1-40 | 129 | 6·2 | 147 | 7·7 | 90 | 4·9 | 92 | 5·5 |
| Percentile 41-80 | 504 | 24·3 | 413 | 21·7 | 416 | 22·7 | 373 | 22·4 |
| Percentile 81-100 | 822 | 39·7 | 653 | 34·3 | 866 | 47·3 | 800 | 48·0 |
| Missing | 618 | 29·8 | 692 | 36·3 | 458 | 25·0 | 402 | 24·1 |
| **NUMBER OF HEALTHCARE PROFESSIONAL PARENTS** |  |  |  |  |  |  |  |  |
| 0 parents | 1788 | 86·3 | 1766 | 92·7 | 1500 | 82·0 | 1289 | 77·3 |
| 1 or 2 parents | 285 | 13·7 | 139 | 7·3 | 330 | 18·0 | 378 | 22·7 |

--

|  | **RADIOLOGY (N=1524)** | | **CARDIOLOGY (N=1370)** | | **OBSTETRICS & GYNAECOLOGY (N=1345)** | | **NEUROLOGY (N=1206)** | |
| --- | --- | --- | --- | --- | --- | --- | --- | --- |
|  | Frequency | Percentage | Frequency | Percentage | Frequency | Percentage | Frequency | Percentage |
| **SEX** |  |  |  |  |  |  |  |  |
| Male | 1006 | 66·0 | 1010 | 73·7 | 444 | 33·0 | 621 | 51·5 |
| Female | 518 | 34·0 | 360 | 26·3 | 901 | 67·0 | 585 | 48·5 |
| **MIGRATION BACKGROUND** |  |  |  |  |  |  |  |  |
| No migration background | 1104 | 72·4 | 943 | 68·8 | 1071 | 79·6 | 998 | 82·8 |
| Turkish or Moroccan | 21 | 1·4 | 45 | 3·3 | 10 | 0·7 | 10 | 0·8 |
| Surinamese, Dutch Caribbean or Indonesian | 75 | 4·9 | 96 | 7·0 | 51 | 3·8 | 35 | 2·9 |
| European | 139 | 9·1 | 118 | 8·6 | 97 | 7·2 | 75 | 6·2 |
| Other | 185 | 12·1 | 168 | 12·3 | 116 | 8·6 | 88 | 7·3 |
| **INCOME PERCENTILE** |  |  |  |  |  |  |  |  |
| Percentile 1-70 | 303 | 19·9 | 309 | 22·6 | 342 | 25·4 | 251 | 20·8 |
| Percentile 71-90 | 345 | 22·6 | 282 | 20·6 | 310 | 23·0 | 298 | 24·7 |
| Percentile 91-100 | 589 | 38·6 | 484 | 35·3 | 462 | 34·3 | 467 | 38·7 |
| Missing | 287 | 18·8 | 295 | 21·5 | 231 | 17·2 | 190 | 15·8 |
| **ASSETS PERCENTILE** |  |  |  |  |  |  |  |  |
| Percentile 1-40 | 100 | 6·6 | 97 | 7·1 | 75 | 5·6 | 62 | 5·1 |
| Percentile 41-80 | 323 | 21·2 | 305 | 22·3 | 281 | 20·9 | 251 | 20·8 |
| Percentile 81-100 | 695 | 45·6 | 576 | 42·0 | 640 | 47·6 | 596 | 49·4 |
| Missing | 406 | 26·6 | 392 | 28·6 | 349 | 25·9 | 297 | 24·6 |
| **NUMBER OF HEALTHCARE PROFESSIONAL PARENTS** |  |  |  |  |  |  |  |  |
| 0 parents | 1174 | 77·0 | 1084 | 79·1 | 1120 | 83·3 | 974 | 80·8 |
| 1 or 2 parents | 350 | 23·0 | 286 | 20·9 | 225 | 16·7 | 232 | 19·2 |

--

|  | **INSURANCE MEDICINE (N=1192)** | | **ORTHOPAEDICS (N=987)** | | **PUBLIC HEALTH MEDICINE (N=870)** | | **OPHTHALMOLOGY (N=856)** | |
| --- | --- | --- | --- | --- | --- | --- | --- | --- |
|  | Frequency | Percentage | Frequency | Percentage | Frequency | Percentage | Frequency | Percentage |
| **SEX** |  |  |  |  |  |  |  |  |
| Male | 698 | 58·6 | 846 | 85·7 | 279 | 32·1 | 424 | 49·5 |
| Female | 494 | 41·4 | 141 | 14·3 | 591 | 67·9 | 432 | 50·5 |
| **MIGRATION BACKGROUND** |  |  |  |  |  |  |  |  |
| No migration background | 864 | 72·5 | 806 | 81·7 | 715 | 82·2 | 549 | 64·1 |
| Turkish or Moroccan | 21 | 1·8 | <10 | blinded | <5 | blinded | 18 | 2·1 |
| Surinamese, Dutch Caribbean or Indonesian | 104 | 8·7 | <40 | blinded | <35 | blinded | 41 | 4·8 |
| European | 63 | 5·3 | 44 | 4·5 | 38 | 4·4 | 108 | 12·6 |
| Other | 140 | 11·7 | 96 | 9·7 | 80 | 9·2 | 140 | 16·4 |
| **INCOME PERCENTILE** |  |  |  |  |  |  |  |  |
| Percentile 1-70 | 443 | 37·2 | 201 | 20·4 | 296 | 34·0 | 197 | 23·0 |
| Percentile 71-90 | 207 | 17·4 | 233 | 23·6 | 180 | 20·7 | 176 | 20·6 |
| Percentile 91-100 | 154 | 12·9 | 410 | 41·5 | 147 | 16·9 | 267 | 31·2 |
| Missing | 388 | 32·6 | 143 | 14·5 | 247 | 28·4 | 216 | 25·2 |
| **ASSETS PERCENTILE** |  |  |  |  |  |  |  |  |
| Percentile 1-40 | 99 | 8·3 | 42 | 4·3 | 44 | 5·1 | 39 | 4·6 |
| Percentile 41-80 | 264 | 22·1 | 213 | 21·6 | 190 | 21·8 | 133 | 15·5 |
| Percentile 81-100 | 380 | 31·9 | 505 | 51·2 | 337 | 38·7 | 397 | 46·4 |
| Missing | 449 | 37·7 | 227 | 23·0 | 299 | 34·4 | 287 | 33·5 |
| **NUMBER OF HEALTHCARE PROFESSIONAL PARENTS** |  |  |  |  |  |  |  |  |
| 0 parents | 1117 | 93·7 | 751 | 76·1 | 796 | 91·5 | 667 | 77·9 |
| 1 or 2 parents | 75 | 6·3 | 236 | 23·9 | 74 | 8·5 | 189 | 22·1 |

--

|  | **RESPIRATORY MEDICINE (N=840)** | | **REHABILITATION MEDICINE (N=710)** | | **GASTRO-ENTEROLOGY (N=694)** | | **DERMATOLOGY & VENEREOLOGY (N=689)** | |
| --- | --- | --- | --- | --- | --- | --- | --- | --- |
|  | Frequency | Percentage | Frequency | Percentage | Frequency | Percentage | Frequency | Percentage |
| **SEX** |  |  |  |  |  |  |  |  |
| Male | 455 | 54·2 | 219 | 30·8 | 407 | 58·6 | 277 | 40·2 |
| Female | 385 | 45·8 | 491 | 69·2 | 287 | 41·4 | 412 | 59·8 |
| **MIGRATION BACKGROUND** |  |  |  |  |  |  |  |  |
| No migration background | 673 | 80·1 | 601 | 84·6 | 537 | 77·4 | 507 | 73·6 |
| Turkish or Moroccan | <10 | blinded | <5 | blinded | <10 | blinded | <5 | blinded |
| Surinamese, Dutch Caribbean or Indonesian | <45 | blinded | <30 | blinded | <20 | blinded | <50 | blinded |
| European | 54 | 6·4 | 34 | 4·8 | 59 | 8·5 | 62 | 9·0 |
| Other | 64 | 7·6 | 47 | 6·6 | 71 | 10·2 | 71 | 10·3 |
| **INCOME PERCENTILE** |  |  |  |  |  |  |  |  |
| Percentile 1-70 | 232 | 27·6 | 186 | 26·2 | 132 | 19·0 | 150 | 21·8 |
| Percentile 71-90 | 184 | 21·9 | 201 | 28·3 | 139 | 20·0 | 158 | 22·9 |
| Percentile 91-100 | 275 | 32·7 | 239 | 33·7 | 303 | 43·7 | 236 | 34·3 |
| Missing | 149 | 17·7 | 84 | 11·8 | 120 | 17·3 | 145 | 21·0 |
| **ASSETS PERCENTILE** |  |  |  |  |  |  |  |  |
| Percentile 1-40 | 52 | 6·2 | 28 | 3·9 | 41 | 5·9 | 27 | 3·9 |
| Percentile 41-80 | 198 | 23·6 | 196 | 27·6 | 143 | 20·6 | 127 | 18·4 |
| Percentile 81-100 | 376 | 44·8 | 341 | 48·0 | 342 | 49·3 | 332 | 48·2 |
| Missing | 214 | 25·5 | 145 | 20·4 | 168 | 24·2 | 203 | 29·5 |
| **NUMBER OF HEALTHCARE PROFESSIONAL PARENTS** |  |  |  |  |  |  |  |  |
| 0 parents | 681 | 81·1 | 579 | 81·5 | 530 | 76·4 | 545 | 79·1 |
| 1 or 2 parents | 159 | 18·9 | 131 | 18·5 | 164 | 23·6 | 144 | 20·9 |

--

|  | **OTORHINOLARYN-GOLOGY (N=628)** | | **UROLOGY (N=543)** | | **PATHOLOGICAL ANATOMY (N=517)** | | **PLASTIC SURGERY (N=418)** | |
| --- | --- | --- | --- | --- | --- | --- | --- | --- |
|  | Frequency | Percentage | Frequency | Percentage | Frequency | Percentage | Frequency | Percentage |
| **SEX** |  |  |  |  |  |  |  |  |
| Male | 418 | 66·6 | 378 | 69·6 | 246 | 47·6 | 264 | 63·2 |
| Female | 210 | 33·4 | 165 | 30·4 | 271 | 52·4 | 154 | 36·8 |
| **MIGRATION BACKGROUND** |  |  |  |  |  |  |  |  |
| No migration background | 499 | 79·5 | 426 | 78·5 | 341 | 66·0 | 286 | 68·4 |
| Turkish or Moroccan | <5 | blinded | <5 | blinded | <10 | blinded | <5 | blinded |
| Surinamese, Dutch Caribbean or Indonesian | <25 | blinded | <25 | blinded | <45 | blinded | <30 | blinded |
| European | 38 | 6·1 | 36 | 6·6 | 59 | 11·4 | 42 | 10·0 |
| Other | 64 | 10·2 | 56 | 10·3 | 70 | 13·5 | 61 | 14·6 |
| **INCOME PERCENTILE** |  |  |  |  |  |  |  |  |
| Percentile 1-70 | 113 | 18·0 | 115 | 21·2 | 131 | 25·3 | 92 | 22·0 |
| Percentile 71-90 | 124 | 19·7 | 117 | 21·5 | 103 | 19·9 | 81 | 19·4 |
| Percentile 91-100 | 267 | 42·5 | 202 | 37·2 | 133 | 25·7 | 153 | 36·6 |
| Missing | 124 | 19·7 | 109 | 20·1 | 150 | 29·0 | 92 | 22·0 |
| **ASSETS PERCENTILE** |  |  |  |  |  |  |  |  |
| Percentile 1-40 | 22 | 3·5 | 21 | 3·9 | 32 | 6·2 | 22 | 5·3 |
| Percentile 41-80 | 114 | 18·2 | 99 | 18·2 | 110 | 21·3 | 69 | 16·5 |
| Percentile 81-100 | 313 | 49·8 | 260 | 47·9 | 194 | 37·5 | 200 | 47·8 |
| Missing | 179 | 28·5 | 163 | 30·0 | 181 | 35·0 | 127 | 30·4 |
| **NUMBER OF HEALTHCARE PROFESSIONAL PARENTS** |  |  |  |  |  |  |  |  |
| 0 parents | 469 | 74·7 | 414 | 76·2 | 446 | 86·3 | 321 | 76·8 |
| 1 or 2 parents | 159 | 25·3 | 129 | 23·8 | 71 | 13·7 | 97 | 23·2 |

--

|  | **RHEUMATOLOGY (N=411)** | | **RADIOTHERAPY (N=403)** | | **GERIATRICS (N=392)** | | **MICROBIOLOGY – BACTERIOLOGY (N=358)** | |
| --- | --- | --- | --- | --- | --- | --- | --- | --- |
|  | Frequency | Percentage | Frequency | Percentage | Frequency | Percentage | Frequency | Percentage |
| **SEX** |  |  |  |  |  |  |  |  |
| Male | 162 | 39·4 | 163 | 40·4 | 90 | 23·0 | 186 | 52·0 |
| Female | 249 | 60·6 | 240 | 59·6 | 302 | 77·0 | 172 | 48·0 |
| **MIGRATION BACKGROUND** |  |  |  |  |  |  |  |  |
| No migration background | 308 | 74·9 | 308 | 76·4 | 340 | 86·7 | 259 | 72·3 |
| Turkish or Moroccan | <5 | blinded | <5 | blinded | <5 | blinded | <5 | blinded |
| Surinamese, Dutch Caribbean or Indonesian | <20 | blinded | <25 | blinded | <15 | blinded | <20 | blinded |
| European | 36 | 8·8 | 38 | 9·4 | 13 | 3·3 | 27 | 7·5 |
| Other | 48 | 11·7 | 32 | 7·9 | 25 | 6·4 | 53 | 14·8 |
| **INCOME PERCENTILE** |  |  |  |  |  |  |  |  |
| Percentile 1-70 | 113 | 27·5 | 94 | 23·3 | 106 | 27·0 | 83 | 23·2 |
| Percentile 71-90 | 85 | 20·7 | 95 | 23·6 | 110 | 28·1 | 73 | 20·4 |
| Percentile 91-100 | 119 | 29·0 | 136 | 33·7 | 132 | 33·7 | 135 | 37·7 |
| Missing | 94 | 22·9 | 78 | 19·4 | 44 | 11·2 | 67 | 18·7 |
| **ASSETS PERCENTILE** |  |  |  |  |  |  |  |  |
| Percentile 1-40 | 25 | 6·1 | 26 | 6·5 | 30 | 7·7 | 25 | 7·0 |
| Percentile 41-80 | 88 | 21·4 | 73 | 18·1 | 109 | 27·8 | 75 | 20·9 |
| Percentile 81-100 | 176 | 42·8 | 189 | 46·9 | 169 | 43·1 | 169 | 47·2 |
| Missing | 122 | 29·7 | 115 | 28·5 | 84 | 21·4 | 89 | 24·9 |
| **NUMBER OF HEALTHCARE PROFESSIONAL PARENTS** |  |  |  |  |  |  |  |  |
| 0 parents | 344 | 83·7 | 324 | 80·4 | 312 | 79·6 | 280 | 78·2 |
| 1 or 2 parents | 67 | 16·3 | 79 | 19·6 | 80 | 20·4 | 78 | 21·8 |

--

|  | **INTELLECTUAL DISABILITY MEDICINE (N=297)** | | **NUCLEAR MEDICINE (N=213)** | | **CLINICAL GENETICS (N=195)** | | **NEUROLOGICAL SURGERY (N=186)** | |
| --- | --- | --- | --- | --- | --- | --- | --- | --- |
|  | Frequency | Percentage | Frequency | Percentage | Frequency | Percentage | Frequency | Percentage |
| **SEX** |  |  |  |  |  |  |  |  |
| Male | 65 | 21·9 | 129 | 60·6 | 33 | 16·9 | 155 | 83·3 |
| Female | 232 | 78·1 | 84 | 39·4 | 162 | 83·1 | 31 | 16·7 |
| **MIGRATION BACKGROUND** |  |  |  |  |  |  |  |  |
| No migration background | 262 | 88·2 | 153 | 71·8 | 168 | 86·2 | 129 | 69·4 |
| Turkish or Moroccan | <5 | blinded | <5 | blinded | <5 | blinded | <5 | blinded |
| Surinamese, Dutch Caribbean or Indonesian | <5 | blinded | <10 | blinded | <5 | blinded | <15 | blinded |
| European | 10 | 3·4 | 20 | 9·4 | 9 | 4·6 | 15 | 8·1 |
| Other | 21 | 7·1 | 29 | 13·6 | 14 | 7·2 | 26 | 14·0 |
| **INCOME PERCENTILE** |  |  |  |  |  |  |  |  |
| Percentile 1-70 | 75 | 25·3 | 54 | 25·4 | 42 | 21·5 | 49 | 26·3 |
| Percentile 71-90 | 87 | 29·3 | 40 | 18·8 | 61 | 31·3 | 37 | 19·9 |
| Percentile 91-100 | 84 | 28·3 | 74 | 34·7 | 74 | 37·9 | 63 | 33·9 |
| Missing | 51 | 17·2 | 45 | 21·1 | 18 | 9·2 | 37 | 19·9 |
| **ASSETS PERCENTILE** |  |  |  |  |  |  |  |  |
| Percentile 1-40 | 13 | 4·4 | 17 | 8·0 | 9 | 4·6 | 22 | 11·8 |
| Percentile 41-80 | 80 | 26·9 | 39 | 18·3 | 42 | 21·5 | 36 | 19·4 |
| Percentile 81-100 | 129 | 43·4 | 89 | 41·8 | 106 | 54·4 | 77 | 41·4 |
| Missing | 75 | 25·3 | 68 | 31·9 | 38 | 19·5 | 51 | 27·4 |
| **NUMBER OF HEALTHCARE PROFESSIONAL PARENTS** |  |  |  |  |  |  |  |  |
| 0 parents | 255 | 85·9 | 168 | 78·9 | 155 | 79·5 | 154 | 82·8 |
| 1 or 2 parents | 42 | 14·1 | 45 | 21·1 | 40 | 20·5 | 32 | 17·2 |

--

|  | **CARDIOTHORACIC SURGERY (N=168)** | | **SPORTS MEDICINE (N=167)** | | **NO REGISTERED SPECIALTY (N=26853)** | |
| --- | --- | --- | --- | --- | --- | --- |
|  | Frequency | Percentage | Frequency | Percentage | Frequency | Percentage |
| **SEX** |  |  |  |  |  |  |
| Male | 143 | 85·1 | 104 | 62·3 | 8966 | 33·4 |
| Female | 25 | 14·9 | 63 | 37·7 | 17887 | 66·6 |
| Missing |  |  |  |  | 1 | 0·0 |
| **MIGRATION BACKGROUND** |  |  |  |  |  |  |
| No migration background | 93 | 55·4 | 146 | 87·4 | 19847 | 73·9 |
| Turkish or Moroccan | <10 | blinded | <5 | blinded | 637 | 2·4 |
| Surinamese, Dutch Caribbean or Indonesian | <20 | blinded | <5 | blinded | 1424 | 5·3 |
| European | 23 | 13·7 | <10 | blinded | 1751 | 6·5 |
| Other | 31 | 18·5 | 11 | 6·6 | 3195 | 11·9 |
| **INCOME PERCENTILE** |  |  |  |  |  |  |
| Percentile 1-70 | 53 | 31·5 | 50 | 29·9 | 4090 | 15·2 |
| Percentile 71-90 | 42 | 25·0 | 45 | 26·9 | 5970 | 22·2 |
| Percentile 91-100 | 33 | 19·6 | 48 | 28·7 | 13669 | 50·9 |
| Missing | 40 | 23·8 | 24 | 14·4 | 3125 | 11·6 |
| **ASSETS PERCENTILE** |  |  |  |  |  |  |
| Percentile 1-40 | 30 | 17·9 | 12 | 7·2 | 2536 | 9·4 |
| Percentile 41-80 | 28 | 16·7 | 41 | 24·6 | 8041 | 29·9 |
| Percentile 81-100 | 51 | 30·4 | 73 | 43·7 | 10402 | 38·7 |
| Missing | 59 | 35·1 | 41 | 24·6 | 5875 | 21·9 |
| **NUMBER OF HEALTHCARE PROFESSIONAL PARENTS** |  |  |  |  |  |  |
| 0 parents | 155 | 92·3 | 133 | 79·6 | 20053 | 74·7 |
| 1 or 2 parents | 13 | 7·7 | 34 | 20·4 | 6801 | 25·3 |

--

# **Appendix 5: Intersectional descriptive statistics belonging to Table 4**

|  | **BSM cohort** |  | **Physicians from the BSM cohort** |  | **Specialists from the BSM cohort** |  |
| --- | --- | --- | --- | --- | --- | --- |
|  | Frequency | Percentage | Frequency | Percentage | Frequency | Percentage |
| Man without migration background | 1072 | 23,8 | 898 | 22,7 | 712 | 23,7 |
| Woman without migration background | 2366 | 52,5 | 2149 | 54,3 | 1696 | 56,6 |
| Man with Turkish/Moroccan MB | 42 | 0,9 | 38 | 1 | 25 | 0,8 |
| Woman with Turkish/Moroccan MB | 53 | 1,2 | 45 | 1,1 | 32 | 1,1 |
| Man with Surinamese/Dutch Caribbean/Indonesian MB | 98 | 2,2 | 74 | 1,9 | 54 | 1,8 |
| Woman with Surinamese/Dutch Caribbean/Indonesian MB | 164 | 3,6 | 146 | 3,7 | 99 | 3,3 |
| Man with European MB | 59 | 1,3 | 52 | 1,3 | 39 | 1,3 |
| Woman with European MB | 109 | 2,4 | 98 | 2,5 | 58 | 1,9 |
| Man with Other MB | 227 | 5 | 188 | 4,8 | 115 | 3,8 |
| Woman with Other MB | 307 | 6,8 | 268 | 6,8 | 169 | 5,6 |

|  | **All physicians in 2021** |  | **All specialists in 2021** |  |
| --- | --- | --- | --- | --- |
|  | Frequency | Percentage | Frequency | Percentage |
| Man without migration background | 24991 | 32,5 | 18600 | 37,2 |
| Woman without migration background | 34335 | 44,7 | 20851 | 41,7 |
| Man with Turkish/Moroccan MB | 547 | 0,7 | 303 | 0,6 |
| Woman with Turkish/Moroccan MB | 686 | 0,9 | 293 | 0,6 |
| Man with Surinamese/Dutch Caribbean/Indonesian MB | 1576 | 2,1 | 1062 | 2,1 |
| Woman with Surinamese/Dutch Caribbean/Indonesian MB | 1919 | 2,5 | 1008 | 2 |
| Man with European MB | 2169 | 2,8 | 1544 | 3,1 |
| Woman with European MB | 2667 | 3,5 | 1539 | 3,1 |
| Man with Other MB | 3809 | 5 | 2593 | 5,2 |
| Woman with Other MB | 4144 | 5,4 | 2162 | 4,3 |
